# Dihydromyricetin regulates KEAP1‐Nrf2 pathways to enhance the survival of ischemic flap

**DOI:** 10.1002/fsn3.4049

**Published:** 2024-02-20

**Authors:** Xianyao Tao, Xiaoyun Pan, Gang Zhao, Mingyu Xue, Yongjun Rui

**Affiliations:** ^1^ Suzhou Medical College of Soochow University Suzhou Jiangsu China; ^2^ Department of Hand Surgery Wuxi Ninth People's Hospital Affiliated to Soochow University Wuxi Jiangsu China

**Keywords:** angiogenesis, apoptosis, dihydromyricetin, ischemic flap, oxidative stress

## Abstract

In clinical flap practice, there are a lot of studies being done on how to promote the survival of distal random flap necrosis in the hypoxic and ischemic state. As a traditional Chinese medicine, dihydromyricetin (DHM) is crucial in preventing oxidative stress and apoptosis in a number of disorders. In this work, we examined the impact of DHM on the ability to survive of ischemia flaps and looked into its fundamental mechanism. Our results showed that DHM significantly increased the ischemic flaps' survival area, encouraged angiogenesis and blood flow, reduced oxidative stress and apoptosis, and stimulated KEAP1‐Nrf2 (Kelch‐like ECH‐associated protein 1‐nuclear factor erythroid 2‐related factor) signaling pathways. Adeno‐associated virus (AAV) upregulation of KEAP1 expression also negated the favorable effects of DHM on flap survival. By activating KEAP1‐Nrf2 signaling pathways, DHM therapy promotes angiogenesis while reducing oxidative stress and apoptosis.

## Graphical abstract

By activating KEAP1‐Nrf2 (Kelch‐like ECH‐associated protein 1‐nuclear factor erythroid 2‐related factor) signaling pathways, dihydromyricetin (DHM) therapy promotes angiogenesis while reducing oxidative stress and apoptosis.

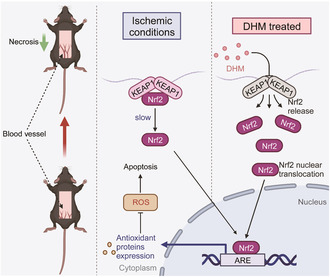

## 1 INTRODUCTION

Flap transplantation is regarded as the most crucial strategy for tissue healing in a variety of reconstructive procedures (Attinger & Colen, 2000; Szeto et al., 2011). Skin abnormalities brought on by severe burns, substantial tumor excision, and automobile accident injuries can all be repaired with skin flap transplantation (Jabir et al., 2015; Ren et al., 2014; Schonauer et al., 2016). Because of its ease, security, and convenience, the random skin flap has become a key reconstructive procedure for tissue reconstruction (Patel, 2022). However, during the transplanting procedure, part of the microvessels supplying the skin flap will be cut, resulting in an ischemic and hypoxic state at the distal end of the skin flap (Lou et al., 2022). In the environment for ischemia as well as hypoxia, a series of pathological chain reactions may occur in the distal flap, for example, the accumulation of peroxide which leads to the increase of oxidative stress level, robust inflammatory reaction, and so on (Chen, Chen, et al., 2023; Li et al., 2022). Cell death, including apoptosis, ferroptosis, and pyroptosis, was further increased with the emergence of these physiological and pathological processes in the flap, which finally aggravated the necrosis of the distal flap (Bali et al., 2021; Yu et al., 2021; Zheng et al., 2022). Finding a method to encourage the survival of the distal portion of the flap is essential for the clinical use of the flap since necrosis at the distal end of the flap restricts its use in medicine.

Angiogenesis is essential for enhancing the flap's survival, particularly in the distal portions that lack a blood supply (He et al., 2021). In order to increase the transport of oxygen and nutrients, the distal flap can quickly create a new network of blood vessels by encouraging angiogenesis (Akhavani et al., 2008). This decreases the anoxic and hypoxic environment as well as the necrosis of the distal flap. Lack of blood flow to the distal flap might result in ischemia and anoxia, which can lead to the accumulation of reactive oxygen species (ROS) (Li, Li, et al., 2023). In addition to causing cell damage and death, the buildup of ROS also causes the generation of additional oxygen‐free radicals, creating a vicious cycle of oxidative stress and cell death (Liu, Sun, et al., 2022; Stockwell et al., 2017). This worsens the hypoxic environment and accelerates the distal flap's necrosis. In order to block this harmful cycle, it is crucial to mitigate the oxidative stress caused by ROS accumulation, thereby reducing the necrosis of the distal flap (Li et al., 2021). Apoptotic pathways can be activated by considerable oxidative stress and damage, which is a key factor in the emergence of distal flap necrosis (Weinzierl et al., 2023). Flap necrosis can result from excessive apoptosis because it kills off too many cells and slows down the healing process. According to earlier research, the distal flap can survive longer when several pharmacological therapies, like Fibroblast Growth Factor 9 (FGF9), are used to suppress apoptosis (Zhang et al., 2023).

Among the many therapeutic strategies adopted to promote the survival of the distal flap, drug injection is a simple and effective treatment (Li et al., 2019; Zhu et al., 2021). Traditional Chinese medicine has utilized dihydromyricetin (DHM), also known as a natural flavonoid component present in several plants, for its many advantageous characteristics (Zhang et al., 2018). DHM can eliminate oxygen‐free radicals and lessen oxidative stress (Guo et al., 2023). It also has exceptional antioxidant stress characteristics. Previous studies have shown that DHM reduces oxidative stress, hence reducing the severity of alcoholic liver injury, cardiac ischemia/reperfusion injury, and diabetic cardiomyopathy (Chen, Zheng, et al., 2023; Silva et al., 2021; Wei et al., 2019). Meanwhile, DHM is widely known for its capacity to affect apoptosis. Previous research has demonstrated that DHM suppresses apoptosis‐related signaling pathways, which lowers neuronal cell death and prevents hepatocyte apoptosis (Liu et al., 2023). DHM also shields human umbilical vein endothelial cells (HUVECs) from damage via the nuclear factor erythroid 2‐related factor/heme oxygenase 1 (Nrf2/HO‐1) signaling pathway (Luo et al., 2017). To mitigate the liver damage caused by ethanol, DHM modifies the Kelch‐like ECH‐associated protein 1 (KEAP1)/Nrf2 pathway (Qiu et al., 2017). Despite the study on DHM's potential protective benefits, including its ability to combat oxidative stress and apoptosis, there is no conclusive scientific evidence that it has a direct impact on flap survival.

Nuclear factor erythroid 2‐related factor 2 (Nrf2), a factor that regulates transcription, contributes an essential part in cellular defense toward oxidative insults through activation of the expression of genes involved in the oxidative stress response (He et al., 2020). Previous experimental studies have demonstrated that the survival of flap can be aided by the activation of Nrf2 via a variety of ways, including pharmacological and genetic strategies (Li, Zhu, et al., 2023). KEAP1, as a negative regulator of the Nrf2 pathway, prevents Nrf2 from activating downstream target genes involved in antioxidant defense and detoxification pathways by binding Nrf2 within the cytoplasm (Liu, Pi, & Zhang, 2022). Numerous diseases, including cancer, cardiovascular illness, and neurological disorders, are tightly correlated with the KEAP1‐Nrf2 network (Chen & Maltagliati, 2018; Kansanen et al., 2013). However, this signaling pathway is rarely studied in skin flaps. Consequently, the purpose of this research is to investigate how DHM affects skin flap survival and to identify any potential underlying mechanisms.

## 2 RESULTS

### 2.1 DHM enhances the survival of ischemic flaps

**FIGURE 1:**
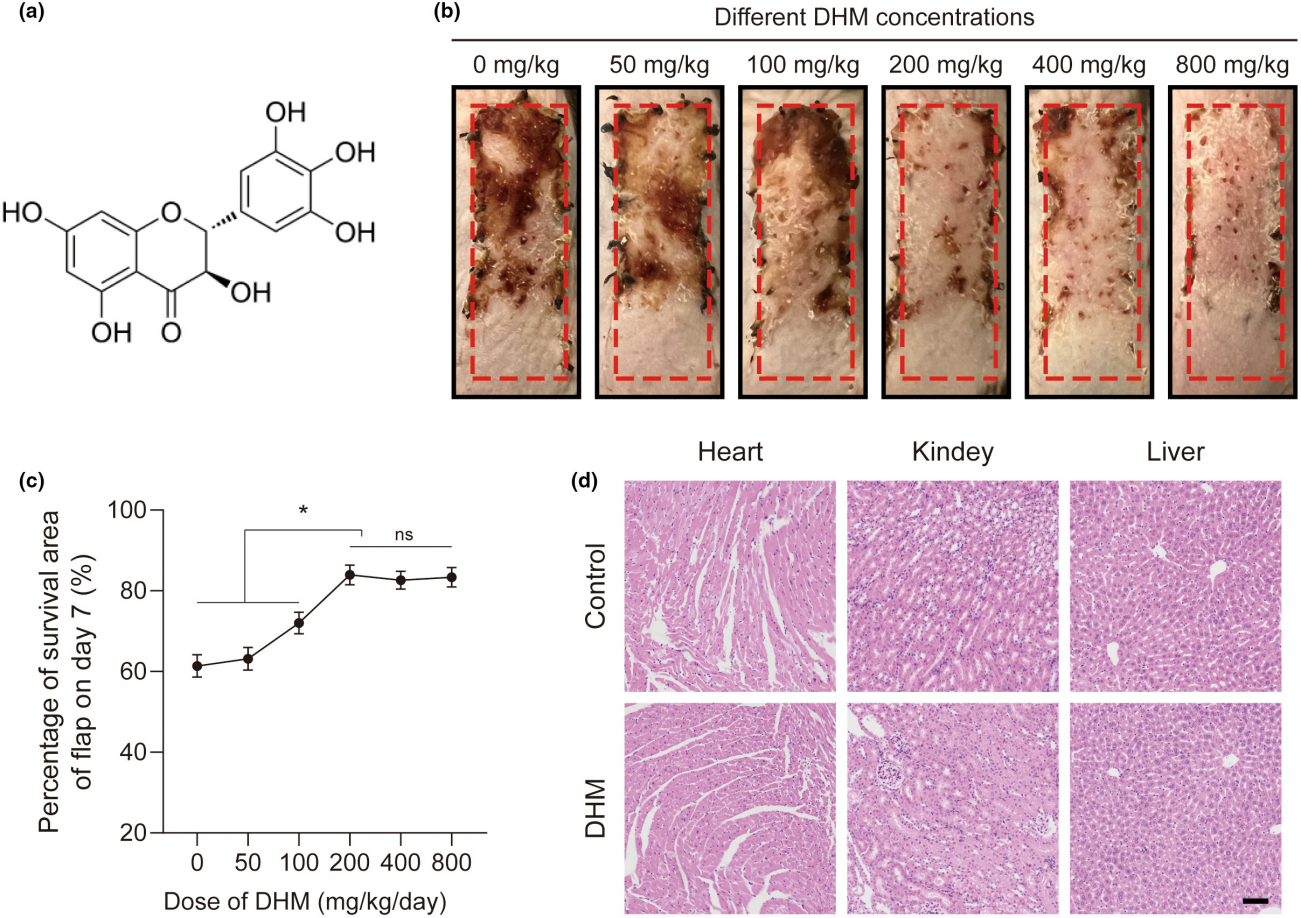
Optimal DHM concentration selection in ischemic flaps. (a) Dihydromyricetin (DHM) chemical structure. (b) Digital images of ischemic flaps after DHM treatment at different concentrations captured on POD7. (c) The dose–response curve showing the optimum dose of DHM (200 mg/kg/day) (*n* = 5). (d) Representative H&E staining of heart, liver, and kidney of mice in the Control group and the DHM group. 50 μm scale bars.

**FIGURE 2:**
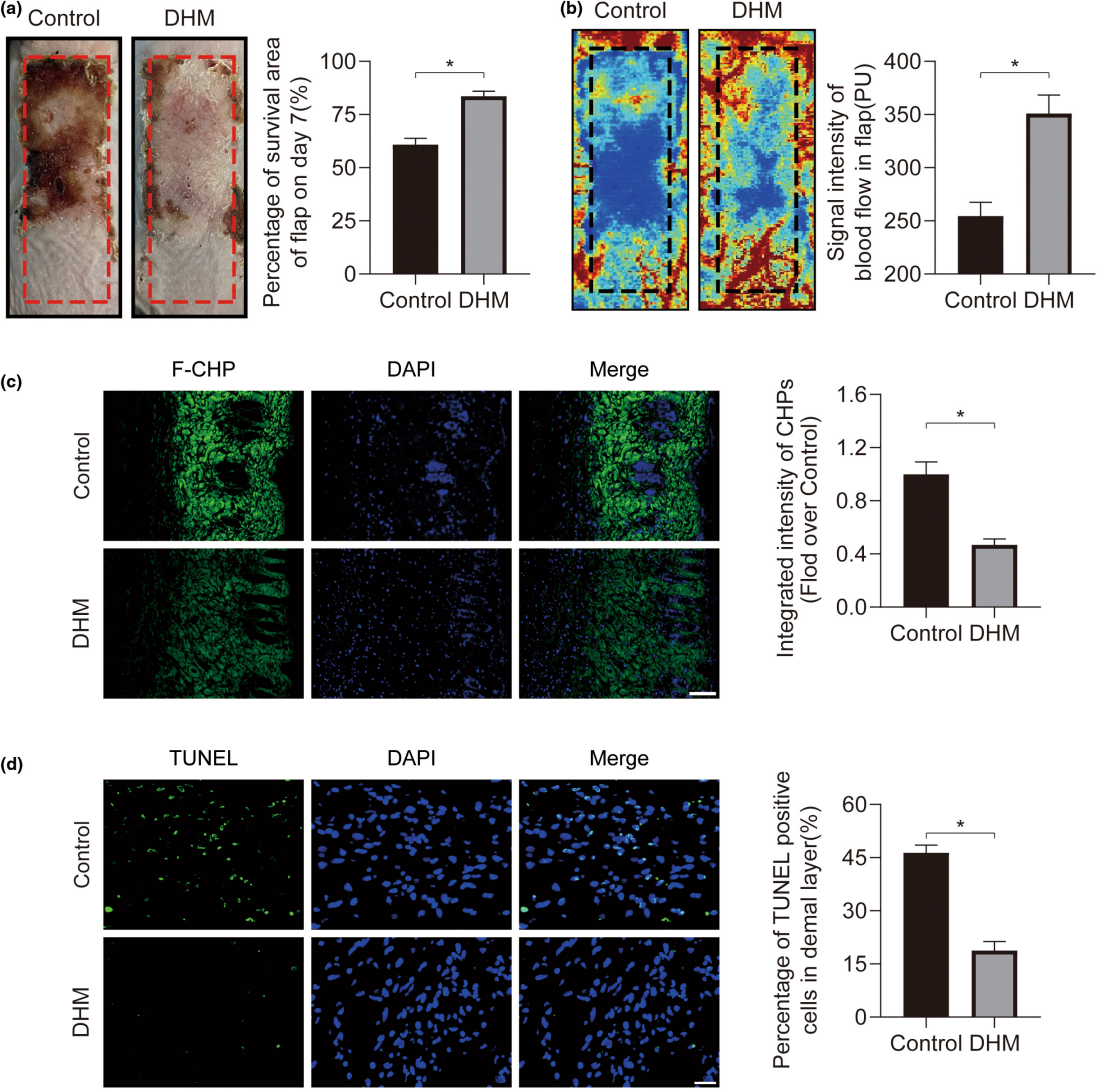
Dihydromyricetin (DHM) enhances the viability of ischemic flaps. (a) Digital photograph of the ischemic flap captured on POD7. Quantification of the percentage of viable flap area on POD7 for both experimental groups (*n* = 5). (b) Visual representation of the subcutaneous blood flow network on POD7. Quantification of blood flow signal intensity in the ischemic flaps for both groups on POD7 (*n* = 5). (c) Damaged collagen in ischemia flaps was found using F‐CHP staining on POD7. 100 μm scale bars. Comparison of the two groups' F‐CHP intensities (*n* = 5). (d) Terminal deoxynucleotidyl transferase dUTP nick end labeling (TUNEL) staining for the identification of dead cells in the ischemic flap on POD7. 50 μm scale bars. Calculation of the proportion of TUNEL‐positive cells in ischemic flaps for both groups (*n* = 5). Error bars are SEM. The band density was normalized and the loading control was β‐actin. Significance: *p < .05, substantially distinct as stated; two‐tailed, unpaired *t*‐test.

On the mouse's back, a random flap model was created to explore ischemia flaps. We observed the ability to survive of skin flaps at various DHM doses in order to determine the ideal dosage. Our findings indicated that the most effective dose of DHM is 200 mg·kg^−1^·day^−1^ (Figure 1b,c). No toxic effects of DHM on heart, liver, and kidney were found in hematoxylin and eosin (H&E) staining (Figure 1d). The distal flap of the Control group underwent the observed alterations, which included a substantial black coloring, rigidity, and necrosis on Day 7. The distal necrotic region greatly decreased within the DHM group compared to the Control group (Figure 2a). Laser Doppler imaging is often used to analyze blood flow, where blue indicates low signal and low blood flow, while red denotes high signal and high blood flow. Statistical analysis showed that blood flow was significantly enhanced when in comparison with the Control group (Figure 2b). In comparison with the Control group, the DHM group demonstrated an obvious decrease in the damaged collagen content of the flap tissue at the Zone II (Figure 2c). The DHM group had fewer dead cells than the Control group, according to TUNEL (terminal deoxynucleotidyl transferase dUTP nick end labeling) staining of the skin flap tissue (Figure 2d). Above all, our findings suggest that DHM has the potential to enhance blood flow and the survival of distal flap.

### 2.2 DHM promotes angiogenesis in ischemic flaps

**FIGURE 3:**
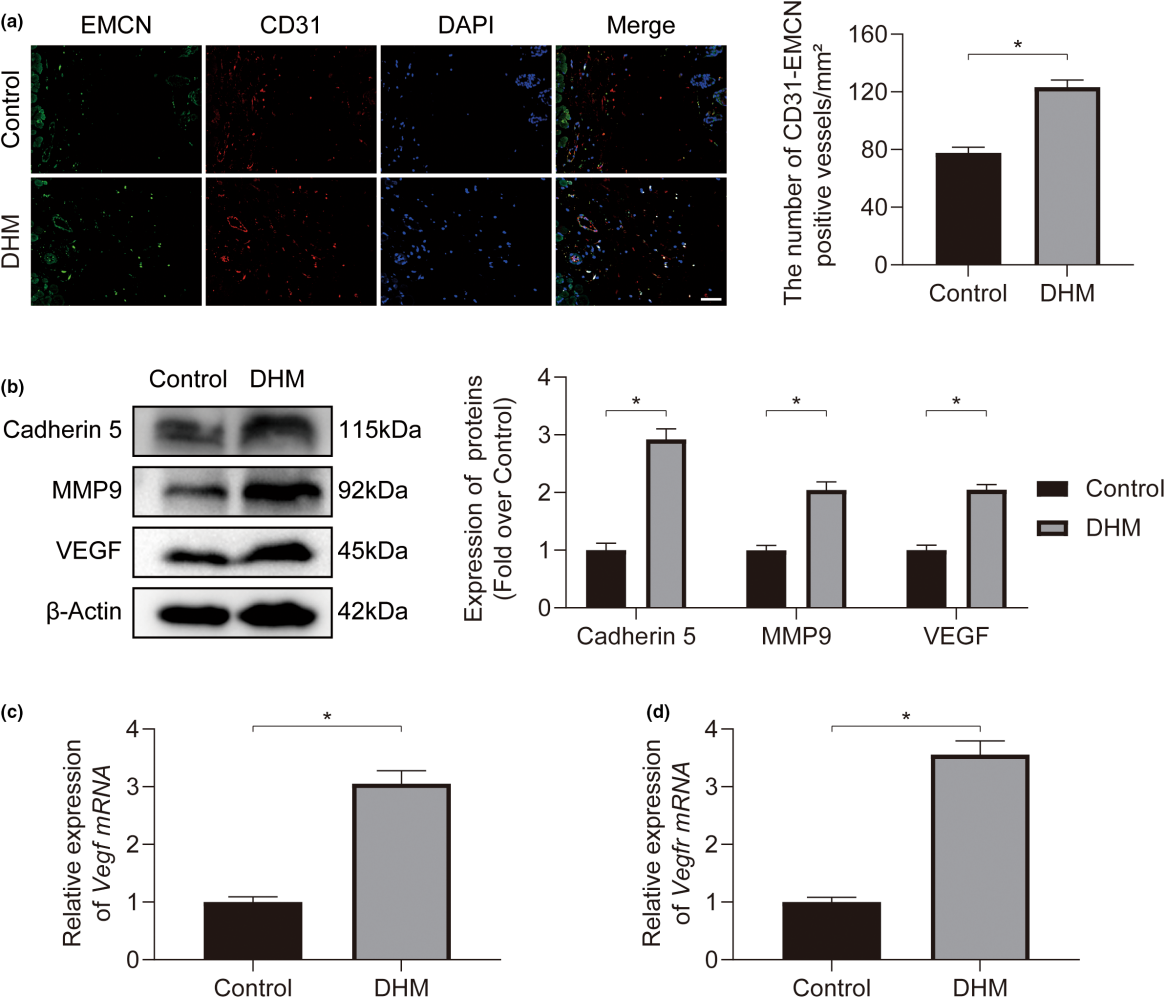
Dihydromyricetin (DHM) promotes angiogenesis in ischemic flaps. (a) Immunofluorescence (IF) staining of CD31 and EMCN in ischemic flaps on POD7. Scale bars: 50 μm. Quantification of CD31/EMCN‐positive blood vessel density between the two groups (*n* = 5). (b) Expression of Cadherin 5, MMP9, and VEGF proteins in ischemic flaps for both groups on POD7. Measurement of the expression of proteins associated with angiogenesis in both groups (*n* = 5). (c) Comparative analysis of relative *Vegf mRNA* expression in ischemic flaps for both groups on POD7 (*n* = 5). (d) Comparative analysis of relative *Vegfr mRNA* expression in ischemic flaps for both groups on POD7 (*n* = 5). Error bars are SEM. The band density was normalized and the loading control was β‐actin. Significance: *p < .05, substantially distinct as stated; two‐tailed, unpaired *t*‐test.

CD31 and Endomucin (EMCN) immunofluorescence (IF) staining was performed to quantify the neovascularization within the Control group and the DHM group. The findings revealed that the DHM group had more CD31 and EMCN‐positive microvessels compared to the Control group (Figure 3a). Cadherin 5 (CDH5), matrix metalloproteinase‐9 (MMP9), and vascular endothelial growth factor (VEGF) levels within the DHM group were shown to be considerably greater than those within the Control group by Western blotting (Figure 3b). Additionally, real‐time quantitative polymerase chain reaction (qPCR) tests showed that *Vegf* and *Vegfr* messenger RNA (mRNA) amounts were increased following DHM treatment (Figure 3c,d). Overall, DHM stimulates angiogenesis in skin flap, as shown by the findings of the immunofluorescence (IF), Western blotting, and real‐time qPCR analyses.

### 2.3 DHM protects ischemic flaps from oxidative stress

**FIGURE 4:**
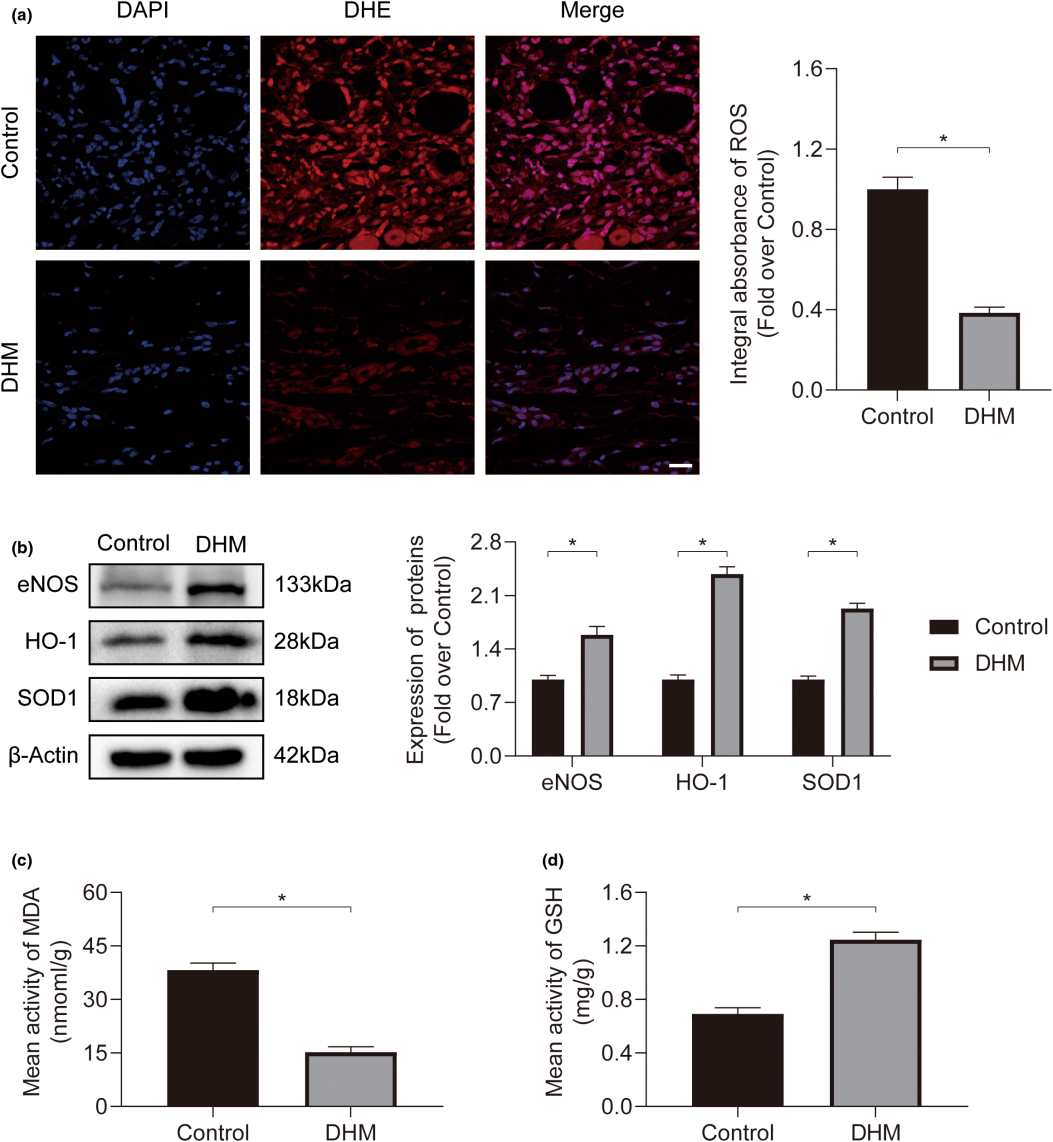
DHM suppresses oxidative stress in ischemic flaps. (a) Ischemic flap frozen sections from both experimental groups on POD7 were subjected to DHE staining. Scale bars: 50 μm. DHE intensity measurements for both groups (*n* = 5). (b) Expression of eNOS, HO‐1, and SOD1 proteins in ischemic flaps for both groups on POD7. Quantification of oxidative stress‐related protein expression for both groups (*n* = 5). (c) Measurement of MDA content in ischemic flaps for both groups on POD7 (*n* = 5). (d) Measurement of GSH content in ischemic flaps for both groups on POD7 (*n* = 5). Error bars are SEM. The band density was normalized and the loading control was β‐actin. Significance: *p < .05, substantially distinct as stated; two‐tailed, unpaired *t*‐test.

To determine the amount of oxygen‐free radicals present in the stromal cells, DHE staining of the skin flap tissue was performed. The findings demonstrated a greater concentration of oxygen‐free radicals had been found within the Control group, in comparison with the DHM group (Figure 4a). Our results of Western blotting, which was detected for the oxidative stress‐related protein in the flaps, demonstrated that the levels of endothelial nitric oxide synthase (eNOS), HO‐1, and superoxide dismutase 1 (SOD1) had risen within the DHM group compared with the Control group (Figure 4b). The content of glutathione (GSH) within the DHM group had been greater compared to that within the Control group, whereas the level of malondialdehyde (MDA) within the DHM group had been significantly lower compared to that within the Control group (Figure 4c,d). These findings show that DHM guards against oxidative damage in ischemia flaps.

### 2.4 DHM protects ischemic flaps from apoptosis

**FIGURE 5:**
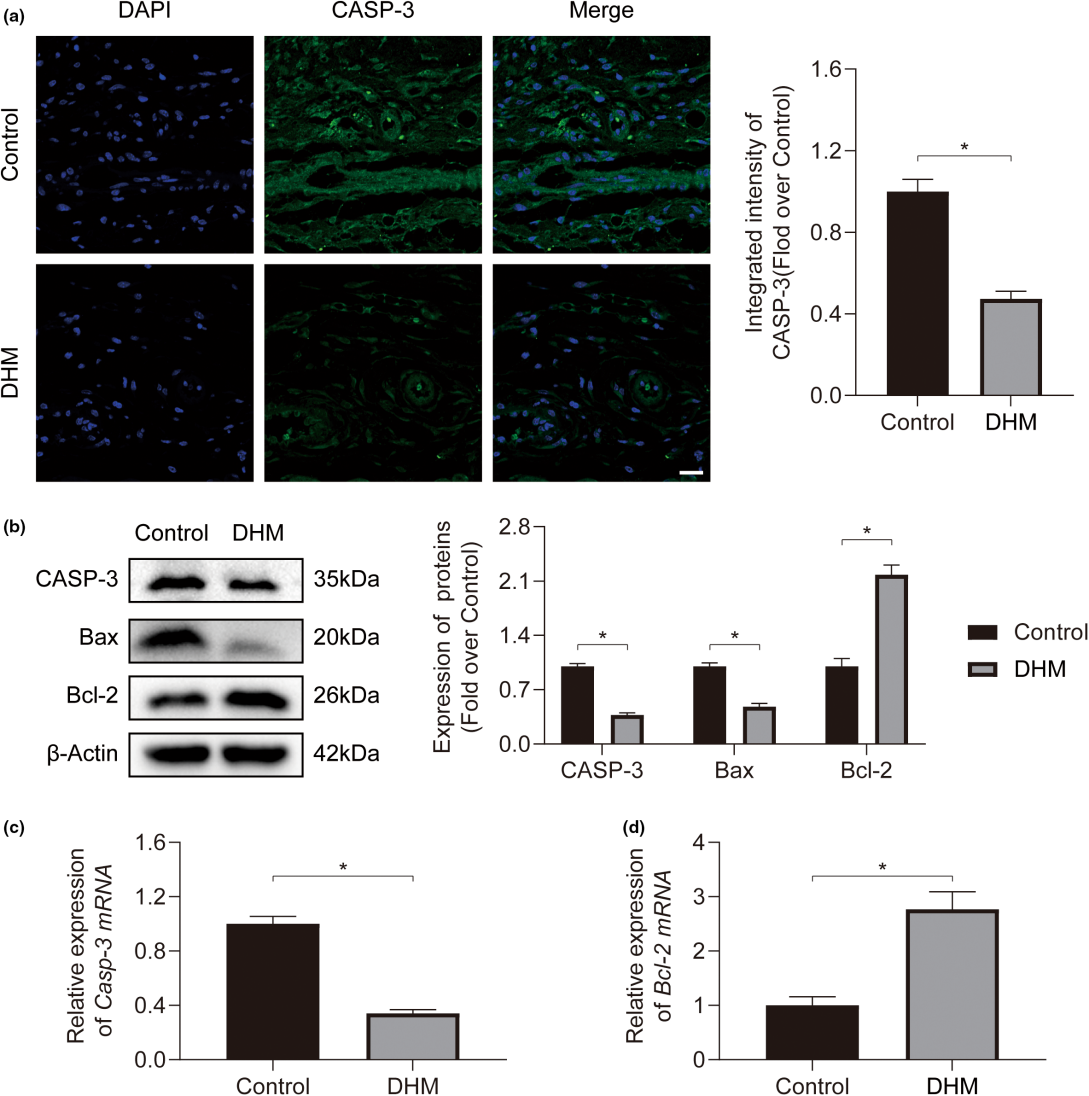
Dihydromyricetin (DHM) mitigates apoptosis in ischemic flaps. (a) Immunofluorescence (IF) staining of CASP‐3 in ischemic flaps on postoperative day 7 (POD7). Scale bars: 20 μm. Quantification of CASP‐3 intensity for both groups (*n* = 5). (b) Expression for CASP‐3, Bax, and Bcl‐2 proteins within ischemic flaps for both groups on POD7. Quantification of apoptosis‐related protein expression for both groups (*n* = 5). (c) Comparative analysis of relative *Casp‐3 mRNA* expression in ischemic flaps for both groups on POD7 (*n* = 5). (d) Comparative analysis of relative *Bcl‐2 mRNA* expression in ischemic flaps for both groups on POD7 (*n* = 5). Error bars are SEM. The band density was normalized and the loading control was β‐actin. Significance: *p < .05, substantially distinct as stated; two‐tailed, unpaired *t*‐test.

Immunofluorescence staining was conducted for Caspase‐3 (CASP‐3) expression to indicate apoptosis level within the ischemic flaps for the Control group and the DHM group. The findings demonstrated that the Control group had greater amounts of CASP‐3 transcription than the DHM group (Figure 5a). Our results from Western blotting, which looked for the apoptosis‐related protein, showed that the DHM group had higher levels of B‐cell lymphoma 2 (Bcl‐2) and lower levels of Bcl‐2‐associated X protein (Bax) and CASP‐3, respectively (Figure 5b). Additionally, a similar pattern to the Western blotting results could be seen in the mRNA expression of CASP‐3 and Bcl‐2 (Figure 5c,d). These findings show that DHM guards ischemic flaps from apoptosis.

### 2.5 DHM inhibits KEAP1 and activates Nrf2 in ischemic flaps

**FIGURE 6:**
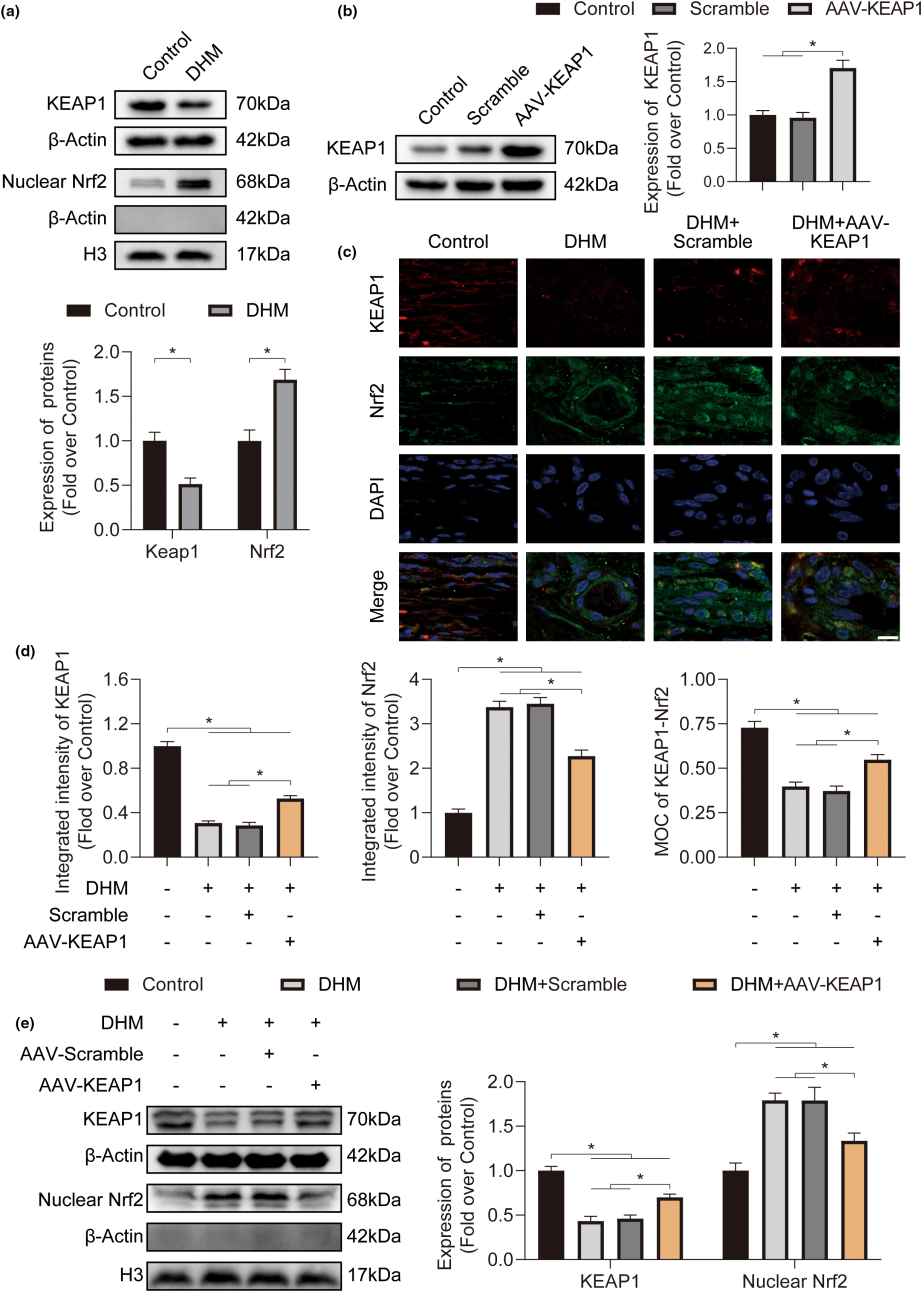
Dihydromyricetin (DHM) activates Nrf2 by suppressing KEAP1 within ischemic flaps. (a) Expression of KEAP1 and nuclear Nrf2 proteins in ischemic flaps for both groups on POD7. Quantification of KEAP1 and nuclear Nrf2 expression between the two groups is presented below (*n* = 5). (b) Kelch‐like ECH‐associated protein 1 (KEAP1) expression in ischemic flaps for all three groups on POD7. The quantified expression of KEAP1‐Nrf2 among these three groups is displayed to the right (*n* = 5). (c) Immunofluorescence (IF) staining for KEAP1 and Nrf2 within ischemic flaps on POD7. Scale bars: 10 μm. (d) Quantification for incorporated intensity in KEAP1 (left), nuclear Nrf2 (middle), and MOCs (Mander's overlap coefficients) of KEAP1‐Nrf2 (right) in ischemic flaps across all four groups (*n* = 5). (e) Expression of KEAP1 and nuclear Nrf2 proteins in ischemic flaps among all four groups on POD7. Quantification of KEAP1 and nuclear Nrf2 expression among all four groups. (*n* = 5). Error bars are SEM. β‐actin and H3 had been used as the loading control and for band density normalization. Significance: *p < .05, substantially distinct as indicated; two‐tailed, unpaired *t*‐test. ANOVA with LSD post hoc testing (categories with equivalent variances) or Dunnett's T3 technique (categories with different variances).

**FIGURE 7:**
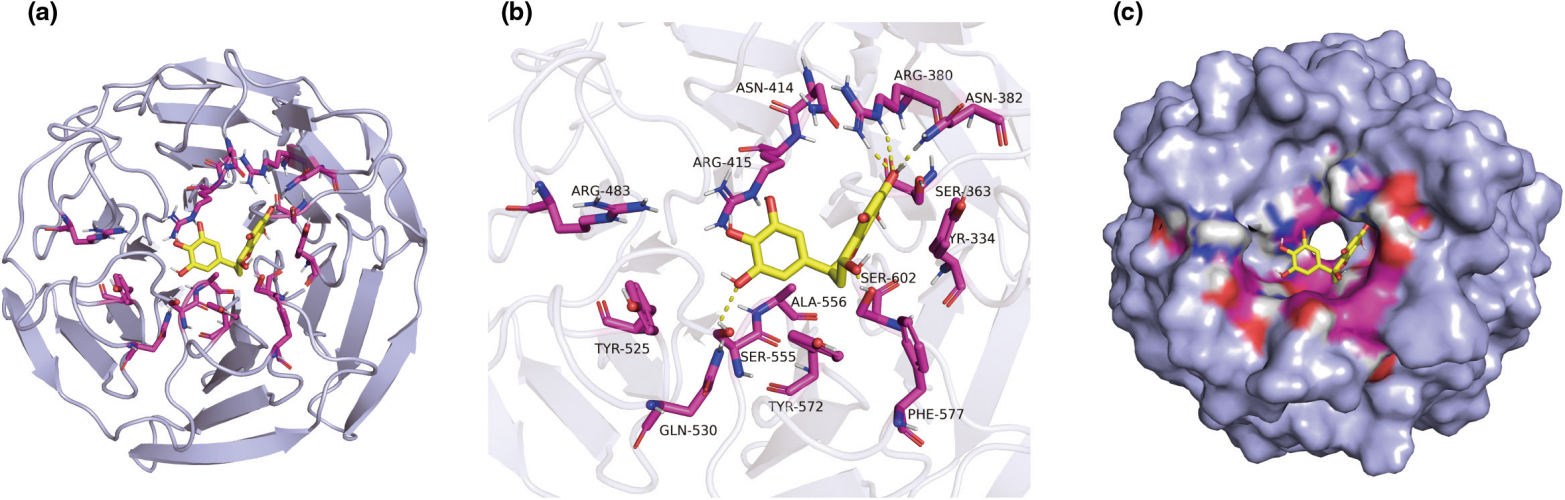
Molecular docking and dynamic simulation between DHM and Keap1‐Nrf2. (a) Kelch‐like ECH‐associated protein 1 (KEAP1) in complex with DHM at the center of the protein. (b) Three‐dimensional (3D) binding model of intermolecular interactions between KEAP1‐Nrf2 and DHM. (c) The space‐filling model of intermolecular interactions between KEAP1‐Nrf2 and DHM.

We assessed the expression of KEAP1 and Nrf2 by Western blotting to look into the inhibition of KEAP1 and activation of Nrf2 in flaps. As a result, optical density (OD) measurements for KEAP1 and Nrf2 were different between the DHM and Control group, with Nrf2's optical density measurement being higher within the DHM group (Figure 6a). To verify the potential of DHM to inhibit oxidative stress in ischemic flaps by triggering the activity of the KEAP1‐Nrf2 process, we enhanced the transcription of KEAP1 using adeno‐associated viruses (AAV)‐KEAP1 (Figure 6b). As demonstrated by the immunofluorescence, the treatment of DHM with AAV‐KEAP1 injection dramatically boosted KEAP1 activity and inhibited Nrf2 nuclear translocation in flaps (Figure 6c,d). In addition, the Western blotting results revealed that the transcription of KEAP1 was higher within the AAV‐KEAP1 + DHM group compared to that within the DHM group and the transcription of Nrf2 had been less within the AAV‐KEAP1 + DHM group compared to that within the DHM group (Figure 6e). As shown in Figure 1a, we used the DHM chemical structure in this analysis. The results of the molecular docking demonstrated a significant binding interaction between DHM and KEAP1 with a binding energy of −7.5 kcal/mol. DHM may have interacted with KEAP1 because it has binding activity with ARG380, ASN382, SER363, TYR334, SER602, SER555, PHE577, ALA556, GLN530, TYR572, TYR525, ARG483, ARG415, and ASN414 in the KEAP1 structure (Figure 7a–c). Above all, these findings show that in ischemia flaps, DHM inhibits KEAP1 and activates Nrf2.

### 2.6 DHM enhances the survival of ischemic flaps by the KEAP1‐Nrf2 pathway

**FIGURE 8:**
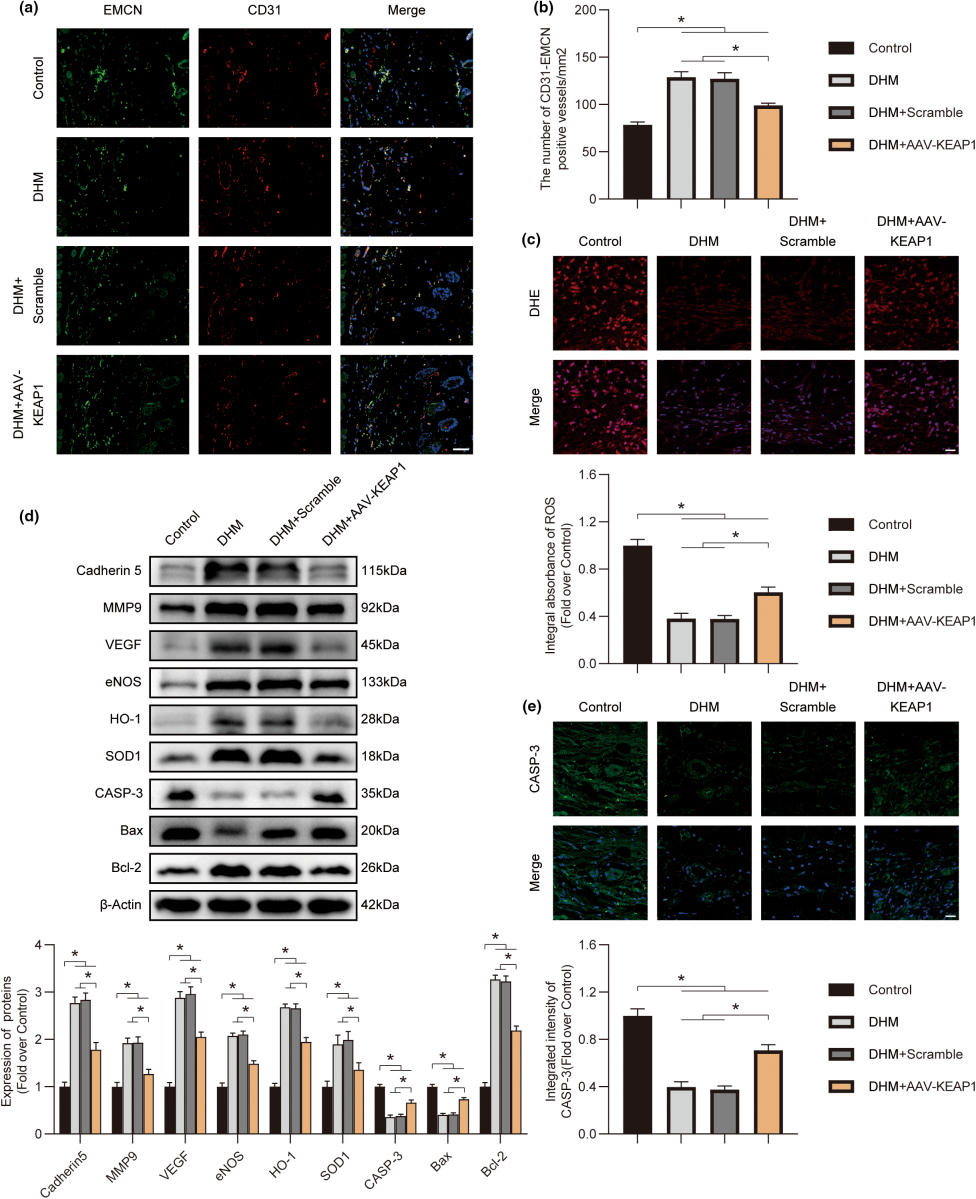
Dihydromyricetin (DHM) alters the KEAP1‐Nrf2 signaling network to promote angiogenesis while reducing oxidative damage and death. (a) Immunofluorescence (IF) staining of CD31 and EMCN in ischemic flaps on POD7. Scale bars: 50 μm. (b) Quantification of CD31/EMCN‐positive blood vessel density across all four groups (*n* = 5). (c) Frozen sections of ischemic flaps from all four groups on POD7 were subjected to DHE staining. Scale bars: 50 μm. Quantified DHE intensity across all four groups is presented below (*n* = 5). (d) Expression of angiogenesis‐, oxidative stress‐, and apoptosis‐related proteins in ischemic flaps among all four groups on POD7. Quantification of expression levels across all four groups is presented below (*n* = 5). (e) Immunofluorescence (IF) staining of CASP‐3 in ischemic flaps on POD7. Scale bars: 20 μm. Quantified CASP‐3 intensity across all four groups is presented below (*n* = 5). Error bars are SEM. The band density was normalized and the loading control was β‐actin. Significance: *p < .05, substantially distinct as specified; Dunnett's T3 technique or ANOVA with LSD post hoc testing (which are equivalent variances for the categories).

The DHM‐treated flaps were treated with AAV‐KEAP1 or Scramble, and levels of angiogenesis, oxidative stress, apoptosis, and the survival of the flap were measured to investigate the role of the KEAP1‐Nrf2 signaling pathway in DHM‐associated angiogenesis, oxidative stress, and apoptosis. As shown by the immunofluorescence, the quantity of CD31 and EMCN‐positive microvessels in the DHM + AAV‐ KEAP1 group had been fewer compared to that within the DHM‐only group and the DHM + Scramble group (Figure 8a,b). Meanwhile, the content of oxygen‐free radicals in stromal cells and the quantity of CASP‐3‐positive cells increased within the DHM + AAV‐ KEAP1 group, in comparison with the DHM‐only group (Figure 8c,e). Western blotting revealed that the expression of Cadherin 5, MMP9, VEGF, Bcl‐2, HO‐1, eNOS, and SOD1 was downregulated, whereas that of Bax and CASP‐3 was upregulated within the DHM + AAV‐ KEAP1 group in comparison with the DHM‐only group (Figure 8d).

**FIGURE 9:**
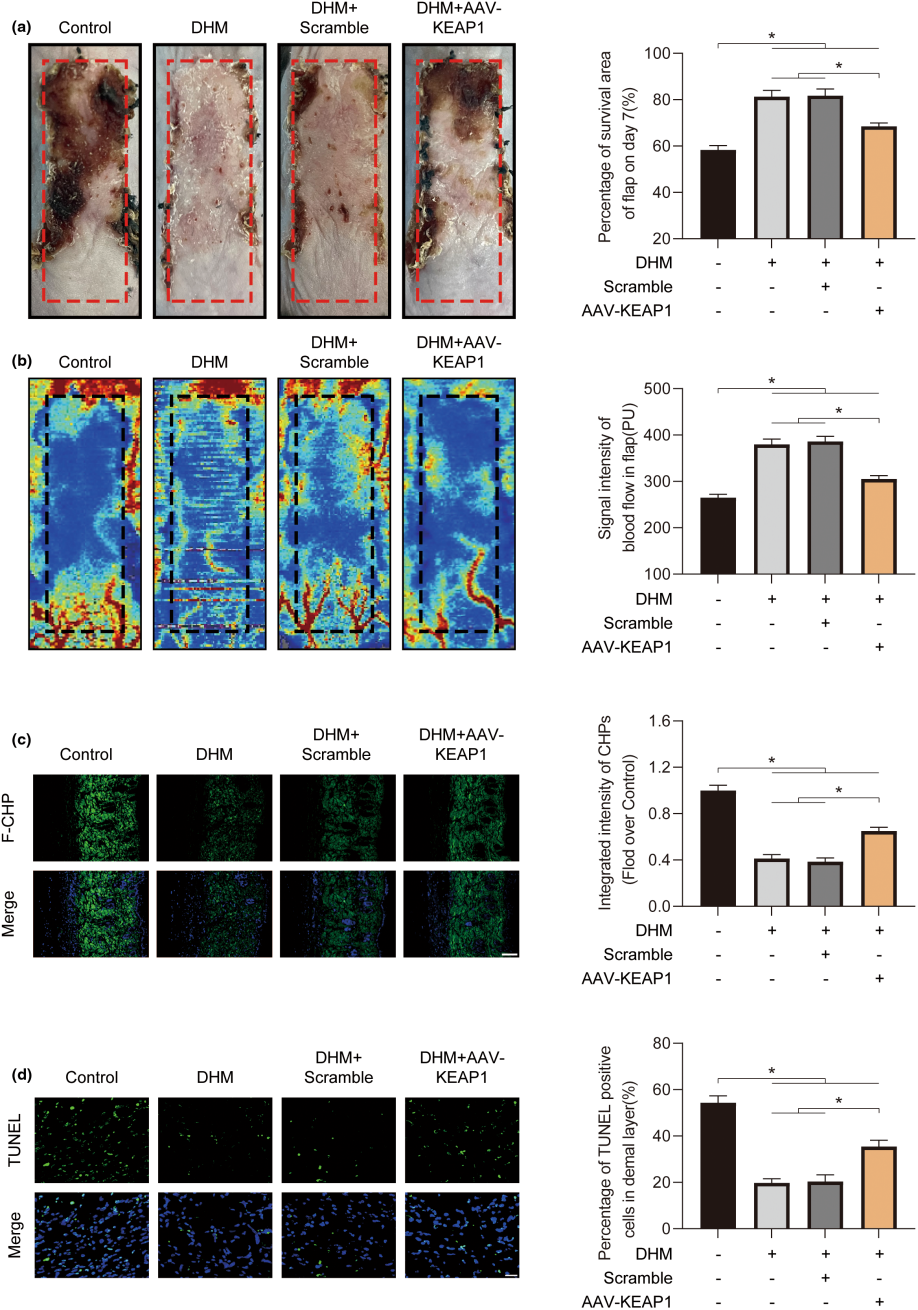
Dihydromyricetin (DHM) enhances the viability of ischemic flaps through the KEAP1‐Nrf2 signaling pathway. (a) Digital photograph of the ischemic flap on POD7. Calculating the proportion of flap area that survived in each of the four groups on POD7 (*n* = 5). (b) Visual representation of the subcutaneous blood flow network on POD7. Quantification of blood flow signal intensity in ischemic flaps across all five groups on POD7 (*n* = 5). (c) Damaged collagen in ischemia flaps was found using F‐CHP staining on POD7. 100 μm scale bars. Measurement of F‐CHP intensity in each of the four groups (*n* = 5). (d) Terminal deoxynucleotidyl transferase dUTP nick end labeling (TUNEL) staining for the identification of dead cells in the ischemic flap on POD7. 50 μm scale bars. The measurement of the proportion of TUNEL‐positive cells in ischemic flaps in all four groups (*n* = 5). Error bars are SEM. Significance: *p < .05, significantly different as indicated; Dunnett's T3 technique or ANOVA with LSD post hoc analysis (which are equivalent variances for the categories).

**FIGURE 10:**
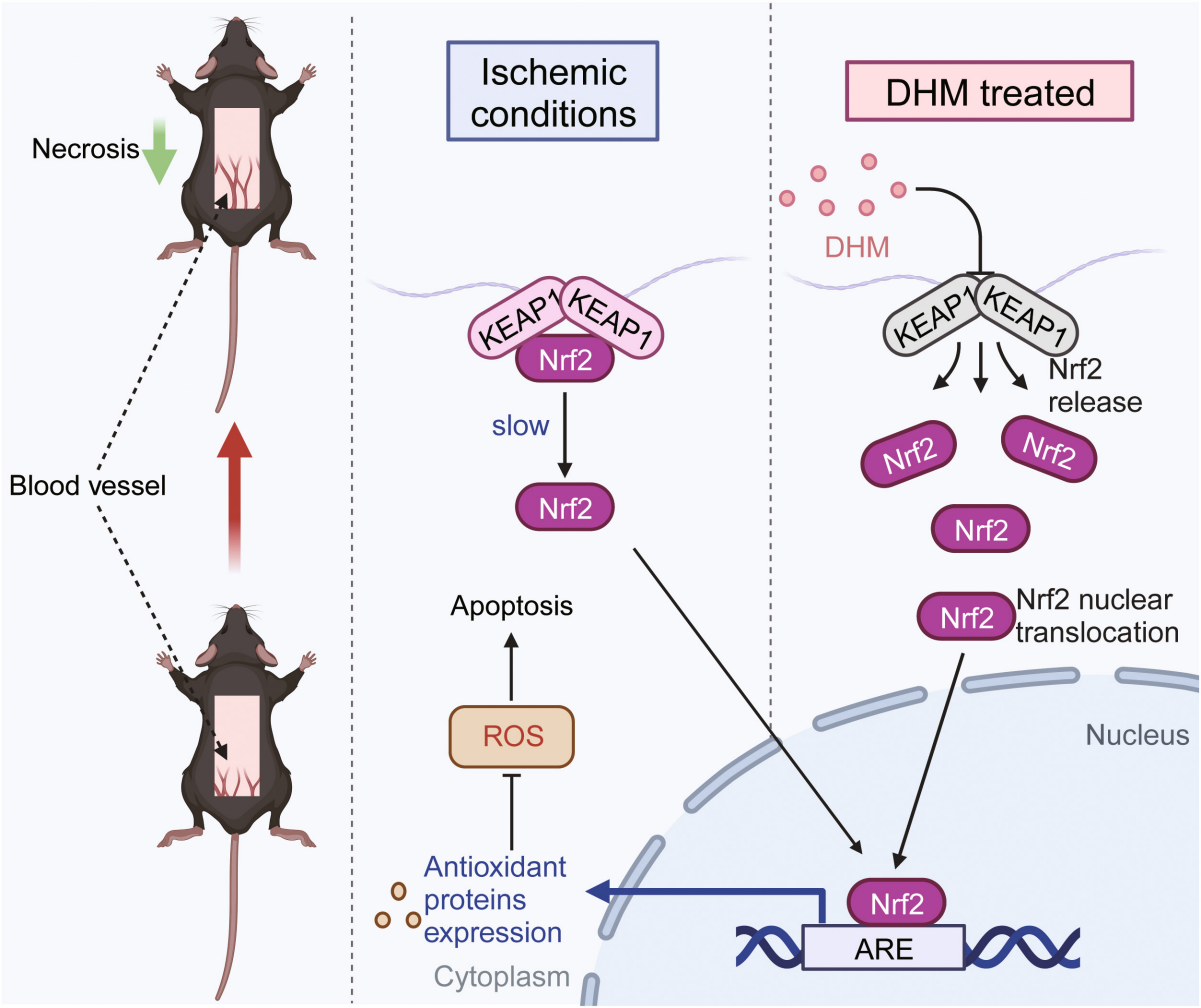
Schematic diagram of the mechanism of DHM promoting ischemic flap survival by inhibiting KEAP1 to upregulate Nrf2 and angiogenesis, while reducing oxidative stress and apoptosis.

Furthermore, the continued existence of ischemic flaps was assessed in each group. On Day 7, there was no discernible difference in the ischemic flap survival between the DHM and DHM + Scramble groups, although the ischemic flap survival area reduced in both the Control group and the DHM + AAV‐KEAP1 group (Figure 9a). The outcomes of the Laser Doppler blood flow (LDBF) were also in line with the survival region of the ischemic flap (Figure 9b). Additionally, in the DHM + AAV‐KEAP1 group as opposed to the DHM, DHM + Scramble groups, the amounts of dead cells rose and the damaged collagen content of the flap increased (Figure 9c,d). Overall, these findings show that DHM improves ischemia flap survival via the KEAP1‐Nrf2 pathway (Figure 10).

## 3 DISCUSSION

Random skin flaps are frequently used to treat large skin defects (Wheeland, 1991). However, the amount to which the flap can be excised and replanted is frequently constrained by the possibility for necrosis (Brinkman et al., 2013). The primary cause of necrosis, which results in increased reactive oxygen species and cell death in the affected tissue, is inadequate blood flow, which causes ischemia and hypoxia of distal tissue (Tu et al., 2021). Ampelopsis grossedentata contains an effective component called DHM, which has a variety of pharmacological characteristics (Sun et al., 2021). Recent research examining the therapeutic benefits of DHM has shown its efficacy in a wide range of illnesses, including diabetic cardiomyopathy, acute renal injury caused by cisplatin, and diabetic cognitive impairment (Chen, Zheng, et al., 2023; Wang et al., 2023; Xu et al., 2023). However, it is still unclear whether DHM can treat ischemia flaps and what are the underlying therapeutic mechanisms. These essential findings of our study presented here had investigated the critical role of DHM in decreasing necrosis in ischemic flaps within a nontoxic and effective dosage range, and explored that DHM effectively suppressed oxidative damage and death through KEAP1‐Nrf2 communication pathway, ultimately leading to enhanced flap survival.

Clinical experience and biological research have established the importance of providing sufficient neovascularization support for blood flow to the distal flap in order to mitigate the hypoxic environment and ischemia (Tanaka et al., 2008; Wu et al., 2019). Similar to this, our findings indicated that DHM significantly increased blood perfusion on LDBF. Both EMCN and CD31 are primarily expressed on the surface of endothelial cells and can be utilized to identify newly formed microvessels. And under the action of DHM, the *Vegf* and *Vegfr* genes and angiogenesis‐related proteins (Cadherin 5, MMP9, and VEGF) were increased in ischemic skin flaps. During ischemia, the supply of oxygen and nutrients to the tissue in the distal flap is reduced, resulting in a disparity between the creation and elimination for ROS, causing ROS accumulation, as well as high levels of MDA (Chen et al., 2018). Both SOD1 and GSH are significant components of cellular defense against oxidative stress (Zhang et al., 2022). Additionally, eNOS and HO‐1 are crucial for preserving endothelial cell functionality and averting oxidative stress (Chiang et al., 2021; Kolluru et al., 2010). It was demonstrated by Western blotting that DHM treatment raised the levels of eNOS, HO‐1, and SOD1 in flaps. In contrast, DHM was found to increase the amount of GSH and decrease the amount of ROS as well as that of MDA in ischemia flaps. The buildup for ROS leads to oxidative damage and a breakdown of DNA and proteins, leading to apoptosis. Previous studies have demonstrated that DHM prevents apoptosis in ischemic stroke cells (Wasan et al., 2022). In normal physiological environment, Bax‐like apoptotic proteins and Bcl‐2‐like anti‐apoptotic proteins maintain a balance, while excessive ROS destroys this balance and increases the production for apoptotic proteins (Hafezi & Rahmani, 2021). By examining the levels of Bax, Bcl‐2, and CASP‐3, we determined the degree of apoptosis that resulted from the administration of DHM within the present investigation. When considered collectively, the data show that the DHM therapy promotes angiogenesis, reduces oxidative stress, and inhibits apoptosis in ischemia flaps.

We subsequently looked into the potential regulatory pathways of angiogenesis, oxidative stress, and apoptosis to clarify how DHM helps flap survive. Previous studies have shown that Nrf2 attaches to antioxidant response elements (AREs) throughout specific gene regulators in the nucleus, thus activating gene transcription for antioxidant response (Tonelli et al., 2018). Only under conditions of oxidative stress can KEAP1 separate from its binding to Nrf2 in the cytoplasm, allowing Nrf2 to go into the nucleus (Kasai et al., 2020). The KEAP1‐Nrf2 signaling pathways perform an essential part within protecting cells from oxidative damage as well as mitigating risk factors for various diseases, such as myocardial ischemia, liver ischemia–reperfusion injury, and retinal vascular injury in diabetes mellitus (Bardallo et al., 2022; Hui et al., 2020; Shen et al., 2019). Our findings indicated that DHM increased and enhanced Nrf2 nuclear translocation while upregulating and downregulating KEAP1. To determine whether the KEAP1‐Nrf2 signaling pathways cause angiogenesis, oxidative stress, and apoptosis caused by DHM, KEAP1 activity was activated using AAV‐KEAP1. We found that the effects of DHM on KEAP1 were greatly reduced by the KEAP1‐AAV vector, and the effects of DHM on Nrf2 nuclear translocation were further reversed. Furthermore, a decrease in the production of angiogenesis, inflammation, as well as proteins associated with apoptosis and a decrease in the flap survival demonstrated that KEAP1‐AAV vector inhibited DHM‐induced angiogenesis, oxidative stress, and apoptosis, suggesting that KEAP1‐Nrf2 signaling pathways may be significant in enhancing the survival of flaps. Consequently, our findings demonstrate that DHM controlled angiogenesis, oxidative stress, and apoptosis through the KEAP1‐Nrf2 signaling pathways.

We believe that this study has the following innovative points: First, DHM has a therapeutic effect in many other diseases, but there are few studies in the field of skin flap. We are the first to apply DHM to the treatment of flap necrosis, providing a new idea for the clinical development of related drugs. Second, the role of the KEAP1‐Nrf2 pathway has not been studied much in the field of skin flap, which provides a new approach for the treatment of distal flap necrosis. Even though these results are encouraging, further study is necessary to look at any further, as yet unidentified, ways that DHM can support flap survival. In addition, more investigations are required to properly comprehend if DHM could be applied in therapeutic settings.

In conclusion, the current investigation showed that DHM was critical for improving flap survivability. Additionally, DHM has the ability to activate the KEAP1‐Nrf2 signaling pathways, which are important for cellular death, angiogenesis, and antioxidant defense. It is plausible to conclude that DHM shows significant promise in clinical practice of treating ischemic skin flaps based on the information reported in this study.

## 4 METHODS

### 4.1 Animals

The Animal Welfare and Use Committee of Wuxi Ninth People's Hospital Affiliated to Soochow University approved each animal experiment performed here in accordance with the Guidelines for the Welfare and Usage of Laboratory Animals of the China National Institutes of Health (KS2023057). Suzhou Medical College of Soochow University's Experimental Animal Center provided C57BL/6J mice (male, 7–8 weeks, mean weight 20–30 g; No. SCXK(JS)2022‐0006). The mice were housed in typical settings with free access to food and water, a temperature range of 21–25°C, relative humidity (RH) between 50% and 60%, and a 12‐h light/dark cycle.

### 4.2 Model with a random skin flap design

Male C57BL/6J mice have been injected intraperitoneally with 50 mg/kg of 1% (w/v) sodium pentobarbital to induce anesthesia. After using an electric shaver to first clip the hair on the back to reveal the skin, depilation was then performed. A surgical flap of 1.5 cm by 4.5 cm had been painstakingly raised beneath the mouse's dorsal fascia using sterile tools. The principal vessel supporting the flap's circulatory system was then severed at the base. Finally, 4‐0 nonabsorbable silk sutures were used to affix the dissected flap to the recipient site.

### 4.3 Groups and treatments

To obtain a dose–response curve, we separately prepared 30 C57BL/6J mice. The mice were randomized into five groups of five mice each, with the dose (mg/kg/day; 0, 50, 100, 200, 400, and 800) applied to each group. Twenty microliters of adeno‐associated virus‐KEAP1 (AAV‐KEAP1) vectors in saline with 9.19 × 10^10^ packaged genomic particles was injected into mice's tail veins 2 weeks before surgery in order to cause the overexpression of KEAP1 in the skin flap tissue. The Control group, referred to as the scrambled Control group, obtained a comparable amount of AAV vector conveying an unfavorable regulate sequence (AAV‐Scramble). Each experimental group consisted of a consistent sample size (*n* = 5). C57BL/6J mice have been separated into one of the six treatment categories at random: Control (*n* = 25), DHM (*n* = 25), AAV‐Scramble (*n* = 5), AAV‐KEAP1 (*n* = 5), DHM + AAV‐Scramble (*n* = 15), and DHM + AAV‐KEAP1 (*n* = 15). DHM was injected intraperitoneally once daily at a dose of 200 mg/kg.

### 4.4 Laser Doppler blood flow (LDBF)

Laser Doppler blood flow (LDBF) imaging was employed to see the random‐pattern skin flap's circulatory system. The mice had been maintained within a controlled, noise‐free environment after postoperative day 7 (POD7) anesthesia. The blood flow to the skin flap was then evaluated using a laser Doppler device. A previously disclosed process was followed by the LDBF assessment technique. In order to assess blood flow, perfusion units (PUs) were computed using MoorLDI Review software (Version 6.1). Three measurements were taken from every mouse, and the average was used. Additional materials offer comprehensive follow‐up experimental procedures.

### 4.5 Materials

The chemicals below were utilized herein: dihydroethidium (DHE) (cat# S0063) from Beyotime Biotechnology (Jiangsu, China); penicillin G (C_16_H_17_KN_2_O_4_S, HPLC ≥ 98.0%, cat# P102194) and carprofen (C_15_H_12_ClNO_2_, HPLC ≥ 98.0%, cat# C153919) from Aladdin (Shanghai, China); Dihydromyricetin (C_15_H_12_O_8_, purity ≥ 98.1%, cat# HY‐N0112) from MedChemExpress (Monmouth Junction, NJ, USA). The Micro Malondialdehyde (MDA) Assay Kit (cat# BC0025) and Micro Reduced GSH Assay Kit (cat# BC1175) were acquired from Solarbio Science & Technology. A BCA tool (cat# 23227), NE‐PER™ nucleus (cat# 78833), and cytoplasmic extraction reagents (cat# 78835) had been provided by Thermo Fisher Scientific (Rockford, IL, USA). Mounting medium with 4′,6‐diamidino‐2‐phenylindole (DAPI)‐aqueous and Fluoroshield (cat# 324 ab104139) were provided by Abcam (Cambridge, UK). The collagen‐associated hybridizing peptide 5‐FAM conjugate (F‐CHP) (cat# FLU300) has been bought through 3Helix (Utah, USA). Cell Signaling Technology (Beverly, MA, USA) provided the primary Caspase‐3 antibodies (CASP‐3; cat# 9662S). Antibodies against KEAP1 (cat# 10503‐2‐AP), NRF2 (cat# 16396‐1‐AP), MMP9 (cat# 10375‐2‐AP), HO‐1 (cat# 10701‐1‐AP), SOD1 (cat# 10269‐1‐AP), Bax (cat# 50599‐2‐Ig), Bcl‐2 (COX2, cat# 26593‐1‐AP), and histone‐H3 (H3; cat# 17168‐1‐AP) were supplied by the Proteintech Group (Chicago, IL, USA). The antibodies against β‐actin (cat# ab213262), VEGFA (cat# ab214424), vascular endothelial (VE) Cadherin (Cadherin; cat# ab205336), eNOS (cat# ab300071), rabbit IgG H&L DyLight® 488 (cat# ab96883), goat anti‐mouse IgG H&L DyLight® 488 (cat# ab96871), and goat anti‐mouse IgG H&L DyLight® 594 (cat# ab96873) were from Abcam (Cambridge, UK). CD31 (cat# GB12063) and Endomucin (EMCN, cat# GB112648) were purchased from Servicebio (Wuhan, China).

### 4.6 AAV vector packaging

The AAV‐KEAP1 vector has been acquired through Shanghai Genechem Co. Ltd. (Shanghai, China). A mouse KEAP1 (NM_016679) sequence was created and then cloned into the pAV‐CMV‐betaGlobin‐MCS‐3Flag‐SV40 PolyA (GV411) plasmid to create the pAV‐CMV‐betaGlobin‐MCS(KEAP1)‐3Flag‐SV40 PolyA construct. The AAV9‐CMV‐betaGlobin‐MCS(KEAP1)‐3Flag‐SV40 and AAV9‐CMV‐betaGlobin‐MSC (scramble)‐3Flag‐SV40 viral vectors were produced by transfecting AAV‐293 cells with pAV‐CMV‐betaGlobin‐MCS(KEAP1)‐3Flag‐SV40, an Ad helper (adenovirus helper plasmid), as well as AAV Rep/Cap expression plasmids. Purification of the virus particles was achieved using the iodixanol gradient approach. The titers of AAV9‐CMV‐betaGlobin‐MCS(KEAP1)‐3Flag‐SV40 and AAV9‐CMV‐betaGlobin‐MSC (scramble)‐3Flag‐SV40 were determined via quantitative PCR and were found to be 9.19E+12 genome copies per milliliter, respectively.

### 4.7 Immunohistochemistry

Necrotic junctional flap tissue from mice has been preserved with 4% paraformaldehyde. The paraffin‐embedded tissue had been dehydrated before being divided into 4‐m pieces. Deparaffinization of the sections in each immunofluorescence (IF) study was carried out using xylene. Following deparaffinization, the tissue underwent rehydration and extraction of antigen using sodium citrate buffer. The sections had been treated using 10% goat serum (Beyotime, C0265) in phosphate‐buffered saline (PBS) (Procell, PB180327) including 0.1% Triton X‐100 (Aladdin, T109027) after cooling to ambient temperature. Subsequently, the sections underwent incubation with primary antibodies overnight at 4°C followed by an hour at ambient temperature via additional antibodies the following day. Nuclear staining was performed using DAPI. The primary antibodies used included CD31 (1:200), EMCN (1:200), CASP‐3 (1:200), KEAP1 (1:200), and Nrf2 (1:200). Goat polyclonal secondary antibody to rabbit immunoglobulin G (IgG), goat anti‐mouse IgG, goat anti‐rabbit IgG, and goat anti‐mouse IgG were the secondary antibodies used. These were H&L DyLight‐488, DyLight‐594, and DyLight‐488. An in situ cell death detection kit had been employed using frozen skin sections, and the manufacturer's instructions were followed. Frozen skin pieces were stained with dihydroethidium (DHE) in accordance with the instructions provided by the manufacturer. The detection of skin collagen degeneration has been carried out using F‐CHP. All measurements were obtained using random sections with five randomly selected fields of view in each section.

### 4.8 Western blotting

Using ice‐cold radioimmunoprecipitation assay (RIPA) lysis buffer (Beyotime, P0013B) enhanced with phenylmethanesulfonyl fluoride (PMSF; Beyotime, ST506) as well as a protease and phosphatase inhibitor cocktail (Beyotime, P1046), flap tissue samples from region II were dissected and homogenized. Cytoplasmic and nuclear protein fractions were abstracted by NE‐PER Nuclear and Cytoplasmic Extraction Kit (Thermo Fisher Scientific, 78835). To obtain tissue lysate, the homogenates were centrifuged at 20,000*g* for 30 min at 4°C. The Omni‐EasyTM Instant BCA Protein Assay Kit was used to measure the protein concentrations. After loading 30 micrograms (mcg) of protein onto 4%–22% SDS‐PAGE (sodium dodecyl sulfate‐polyacrylamide gel electrophoresis) gels, the protein was subsequently moved into polyvinylidene fluoride (PVDF) membranes (Millipore). When primary antibodies were applied, the PVDF membranes had been diluted using 5% skim milk (BD Biosciences, 232100) and incubated at 4°C for 15 h. The membranes were followed by treatment with horseradish peroxidase (HRP)‐conjugated secondary antibodies at room temperature for 1.5 h. An Omni‐ECL Pico Light Chemiluminescence Kit (EpiZyme, SQ201) was utilized to identify protein bands and a ChemiDoc Imaging System (Bio‐Rad) was employed to display the results. The intensity of Western blotting was quantified using Image Lab 3.0 software (Bio‐Rad, Hercules, CA, USA). The following proteins were targeted by the main antibodies in the study: KEAP1 (1:1000), Nrf2 (1:1000), Cadherin 5 (1:1000), MMP9 (1:1000), HO‐1 (1:1000), VEGF (1:1000), eNOS (1:1000), SOD1 (1:1000), β‐actin (1:1000), histone H3 (1:1000), CASP‐3 (1:1000), Bax (1:1000), and Bcl‐2 (1:1000).

### 4.9 qPCR

The total RNA extraction protocol has been previously described. The quantitation process involved a two‐phase chemical reaction: reverse transcription (RT) and polymerase chain reaction (PCR). For individual RT reactions, 0.5 μg of RNA, 2 μL of 5 × TransScript All‐in‐One First‐Strand complementary (cDNA) Synthesis SuperMix for qPCR and 0.5 μL of genomic deoxyribonucleic acid (gDNA) Remover (totaling 10 μL) had been employed. Applied BioSystems' GeneAmp® 9700 PCR System (42°C over 15 min and then a 5‐second denaturation step at 85°C) was used to conduct the experiment. The 10‐L RT reaction mix had been diluted 10 times with nuclease‐free water and incubated at −20°C.

Real‐time PCR has been performed employing the LightCycler® 480 Real‐time PCR Instrument II (Roche, Switzerland) containing a PCR mix volume for 10 μL, 1 μL for cDNA, 5 μL for 2 × PerfectStartTM Green qPCR SuperMix (TransGen Biotech Co., AQ601), 0.2 μL for forward primer, 0.2 μL for reverse primer, and 3.6 μL for nuclease‐free water. The reaction involved an initial denaturation step at 94°C for 0.5 min (Roche, 04729749001), then 45 cycles of 5 s at 94°C and 30 s at 60°C. To ensure that the intended PCR product had been amplified, each material was examined in triplicate and a melting curve assay was run.

The primer sequences used within this study were produced by GeneChem according to the mRNA sequences received through the National Center for Biotechnology Information (NCBI): *Vegf 5′‐CACTGGACCCTGGCTTTACTG‐3′* (forward), *5′‐CTCAATCGGACGGCAGTAGC‐3′* (reverse); *Vegfr5′‐ CCGCCTCTGTGGGTTTGC‐3′* (forward), *5′‐GCCGCATTCAGTCACCAATAC‐3′* (reverse); *Casp‐3 5′‐ GCTGGACTGTGGCATTGAGA‐3′* (forward), *5′‐ TCCAGGAATAGTAACCAGGTGC‐3′* (reverse); *Bcl‐2 5′‐ GTGTGGAGAGCGTCAACAGG‐3′* (forward), *5′‐TATAGTTCCACAAAGGCATCCCAGC‐3′* (reverse);, *Actb 5′‐CTACCTCATGAAGATCCTCACCGA‐3′* (forward), *5′‐TTCTCCTTAATGTCACGCACGATT‐3′* (reverse). The target mRNAs and target microRNAs (miRs) were then adjusted to the level of actin beta (Actb) mRNA. Quantitative PCR (qPCR) analyses were performed using the 2−ΔΔCt method.

### 4.10 Histological examination

Tissue samples were gathered from heart, liver, and kidney in each group after animals were euthanized. Then, tissue samples were fixed in 4% paraformaldehyde for 24 h, and the paraffin‐embedded tissue had been dehydrated before being divided into 4‐m pieces. The microscopic state of tissues was observed by a microscope (Nikon, Tokyo, Japan).

### 4.11 GSH measurements and MDA content

We assessed the amounts of malondialdehyde (MDA) in the Zone II tissue using the Micro MDA Assay Kit (Solarbio, BC0025). Additionally, the Micro Reduced GSH Assay Kit (Solarbio, BC1175) was used to measure the GSH content in the wounded tissue at the Zone II.

### 4.12 Molecular modeling

Kelch‐like ECH‐associated protein 1‐nuclear factor erythroid 2‐related factor 2 (KEAP1‐Nrf2) (PDB ID: 2FLU) was derived from the protein database (https://www.rcsb.org/structure). The molecular makeup of the DHM has been derived via the PubChem database (https://pubchem.ncbi.nlm.nih.gov/). AutoDock Vina v.1.2.0 software has been employed to carry out molecular simulation connecting between the goal protein and the ligand molecule, and PyMol 2.1 software was used to visualize the binding effect.

### 4.13 Statistics

SPSS Statistics 22 (IBM, USA) was used to conduct the statistical evaluation. The standard deviation of the mean (SEM) is used to present all data. All of the data given in this study were normalized to a control group in order to take into consideration potential sources of unintended variation. Analysis for variance (ANOVA) was employed to assess differences among four or five groups, followed by post hoc tests using the least significant difference (LSD) method (assuming equivalent variances) and Dunnett's T3 method (if equivalent variances had not been assumed). Independent sample *t*‐tests had been conducted to identify group‐specific distinctions. Statistical significance was denoted by *p* < .05, the cutoff value.

## AUTHOR CONTRIBUTIONS

Yongjun Rui and Mingyu Xue designed research. Xianyao Tao performed research. Xianyao Tao, Xiaoyun Pan, and Gang Zhao analyzed data. Xianyao Tao wrote the paper. Gang Zhao, Mingyu Xue, and Yongjun Rui revised the paper.

## CONFLICT OF INTEREST STATEMENT

No potential conflict of interest was reported by the author(s).

## ETHICS STATEMENT

The Animal Welfare and Use Committee of Wuxi Ninth People’ s Hospital Affiliated to Soochow University approved each animal experiment performed here in accordance with the Guidelines for the Welfare and Usage of Laboratory Animals of the China National Institutes of Health (KS2023057).

## CONSENT FOR PUBLICATION

All authors agree to publish.

## Data Availability

Additional data collected during this study are available from the corresponding author upon reasonable request.
